# The Parliamentary Inquiry into Mitochondrial Donation Law Reform (Maeve’s Law) Bill 2021 in Australia: A Qualitative Analysis

**DOI:** 10.1007/s11673-023-10257-4

**Published:** 2023-08-02

**Authors:** Jemima W. Allen, Christopher Gyngell, Julian J. Koplin, Danya F. Vears

**Affiliations:** 1 Monash University, Wellington Rd, Clayton, Australia; 2 Biomedical Ethics Research Group, Murdoch Children’s Research Institute, Parkville, Australia; 3 Department of Paediatrics, University of Melbourne, Melbourne, Australia; 4 Melbourne Law School, University of Melbourne, Parkville, Australia; 5 Department of Public Health and Primary Care, Center for Biomedical Ethics and Law, Leuven, Belgium

**Keywords:** Bioethics, Mitochondrial donation, Gene technology, Gene technology regulation, Technological and regulatory advances, Medical technology legislation

## Abstract

Recently, Australia became the second jurisdiction worldwide to legalize the use of mitochondrial donation technology. The *Mitochondrial Donation Law Reform (Maeve’s Law) Bill 2021* allows individuals with a family history of mitochondrial disease to access assisted reproductive techniques that prevent the inheritance of mitochondrial disease. Using inductive content analysis, we assessed submissions sent to the Senate Committee as part of a programme of scientific inquiry and public consultation that informed drafting of the Bill. These submissions discussed a range of bioethical and legal considerations of central importance to the political debate. Significantly, submissions from those with a first-hand experience of mitochondrial disease, including clinicians and those with a family history of mitochondrial disease, were in strong support of this legislation. Those in support of the Bill commended the two-staged approach and rigorous licencing requirements as part of the Bill’s implementation strategy. Submissions which outlined arguments against the legislation either opposed the use of these techniques in general or opposed aspects of the implementation strategy in Australia. These findings offer a window into the ethical arguments and perspectives that matter most to those Australians who took part in the Senate inquiry into mitochondrial donation. The insights garnered from these submissions may be used to help refine policy and guidelines as the field progresses.

## Introduction

On Wednesday March 30, 2022, Australia became the second jurisdiction worldwide to legalize the use of mitochondrial donation technology. The *Mitochondrial Donation Law Reform (Maeve’s Law) Bill 2021* will allow individuals with a family history of mitochondrial disease to access assisted reproductive techniques that prevent the inheritance of mitochondrial disease.

Mitochondrial disease refers to several distinct conditions that occur due to dysfunctional mitochondria. It can affect any organ in the body, and symptoms can begin at any point in the lifespan. In Australia, approximately one child per week is born with a severely disabling form of mitochondrial disease that can cause premature death. Mitochondrial disorders can be categorized into those caused by mutations in either the mitochondria’s own DNA (mitochondrial DNA; mtDNA) or in nuclear DNA (nDNA). While nDNA is inherited from both parents, mtDNA is maternally inherited. Half of the cases of mitochondrial disease are caused by maternally inherited mutations in mtDNA. Mitochondrial donation only affects cases where genetic mutations are in mtDNA; it does not prevent the transmission of nDNA variants.

Mitochondrial donation is a form of in vitro fertilization (IVF) that essentially allows mtDNA from one individual to be combined with the nDNA of another in a single cell (usually an oocyte or zygote). The technology provides a mechanism for many individuals with a family history of mitochondrial disease to avoid passing this genetic risk onto their children. There are several techniques that may be used in mitochondrial donation. The main two are pronuclear transfer (PNT), where the nDNA is transferred after fertilization, and maternal spindle transfer (MST), in which nDNA is transferred before fertilization. Mitochondrial donation is ethically controversial, and many groups and individuals oppose its legalization and use. The controversial nature of this technology partly explains why only the United Kingdom and Australia have legalized mitochondrial donation and only after extensive review and consolation process.

The decision to legalize mitochondrial donation in Australia comes after years of public debate and scientific scrutiny into this issue. In 2018, the Australian Senate conducted an inquiry into the implementation of mitochondrial donation technology in the Australian context, resulting in recommendations for further community consultation and scientific review. This task was taken up by the NHMRC, which in 2019 ran a significant programme of community consultation activities, and ultimately by the Department of Health, which in early 2021 held a community consultation on a potential model for mitochondrial donation in Australia. These processes resulted in the development of *the Mitochondrial Donation Law Reform (Maeve’s Law) Act*, which was drafted and introduced into the House of Representatives on 24th March 2021 as a conscience vote.

On 24 June 2021, the Senate referred the Bill to the Senate Community Affairs Legislation Committee, which invited submissions from key organizations and members of the public. The Bill was ultimately re-introduced to Parliament in November 2021 and passed in March 2022. The ethical implications of mitochondrial donation were of central importance throughout these political debates and processes.

Public consultation sometimes provides evidence that support or rebut ethical concerns and can help identify novel challenges or benefits of a technology. Use of public opinion also ensures that future ethical analysis is targeted to areas that are most prominent and contested. Reflection of this ethical analysis also provides interesting insight into the types of individuals and groups most engaged with the political processes which underpin legislation on controversial bioethical issues. The public consultation phases of Maeve’s law provide a resource for further analysis of the ethics of mitochondrial donation. They offer a window into the ethical ideas and perspectives that matter most to those Australians who took part in the Senate inquiry into mitochondrial donation.

In this paper, we systematically analysed all fifty-six submissions and three supplementary submissions received by the Senate Community Affairs Legislation Committee in 2021 in order to identify the types of arguments for and against the proposed *Mitochondrial Donation Law Reform (Maeve’s Law) Bill 2021*. In this way, we were able to identify some of the issues most pertinent to this controversial debate.

## Method

We used a qualitative methodology and purposively sampled the fifty-six submissions received by the Community Affairs Legislation Committee for inquiry as our dataset. Our inclusion criteria were submissions received by the Senate Committee on or before July 16, 2021. This was an all-inclusive assessment. These submissions, written by a number of different Australian and International stakeholders, discussed arguments relating to the introduction of mitochondrial donation in Australia and are publicly available on the Parliament of Australia website.

To analyse the dataset, we first identified key characteristics of each of the submissions, including the nature of the person or organization who made the submission, research and clinical experience in mitochondrial disease, and whether they opposed Maeve’s Law, supported the Bill without any further amendments, or supported it with minor amendments.

We then used Inductive Content Analysis (ICA) to analyse the content of the submissions (Vears and Gillam 2022). An inductive approach to this analysis was undertaken to ensure that the full meaning and nuance of the arguments were appreciated. Given the dataset consisted of written submissions and the analysis of their arguments, this approach was the most appropriate to best represent the data. Codes were developed from the submission texts, rather than predetermined. These codes corresponded to the arguments for and against the introduction of mitochondrial donation in Australia presented in the submissions. An iterative approach was undertaken such that, with each subsequent reading of the submissions, the coding became more refined through comparing, grouping, and sub-dividing groups of codes into categories. Each submission was coded at least twice; JA coded all submissions and DV, JK and CG each coded a subset. Cross reviews of the coding scheme minimized potential subjectivity when analysing the arguments. Any discrepancies were discussed as a group until consensus was reached.

## Results

A total of fifty-six public submissions were received during the Senate inquiry in July 2021. Of these, thiry-nine submissions supported the introduction of the *Mitochondrial Donation Law Reform (Maeve’s Law) Bill 2021* in Australia and seventeen opposed the Bill. Of those submissions in support, twenty-three supported Maeve’s Law without any further amendments and sixteen supported the legislation with minor amendments, which generally corresponded with requesting specific expertise on the NHMRC Licensing Committee and appropriate regulatory oversight. Of those that opposed the Bill, most did not propose amendments. However, four of the submissions proposed major amendments that would fundamentally alter the implementation of Maeve’s Law as it currently stands, such as removal of Pronuclear Transfer (PNT) from the list of acceptable techniques.

**Fig. 1:**
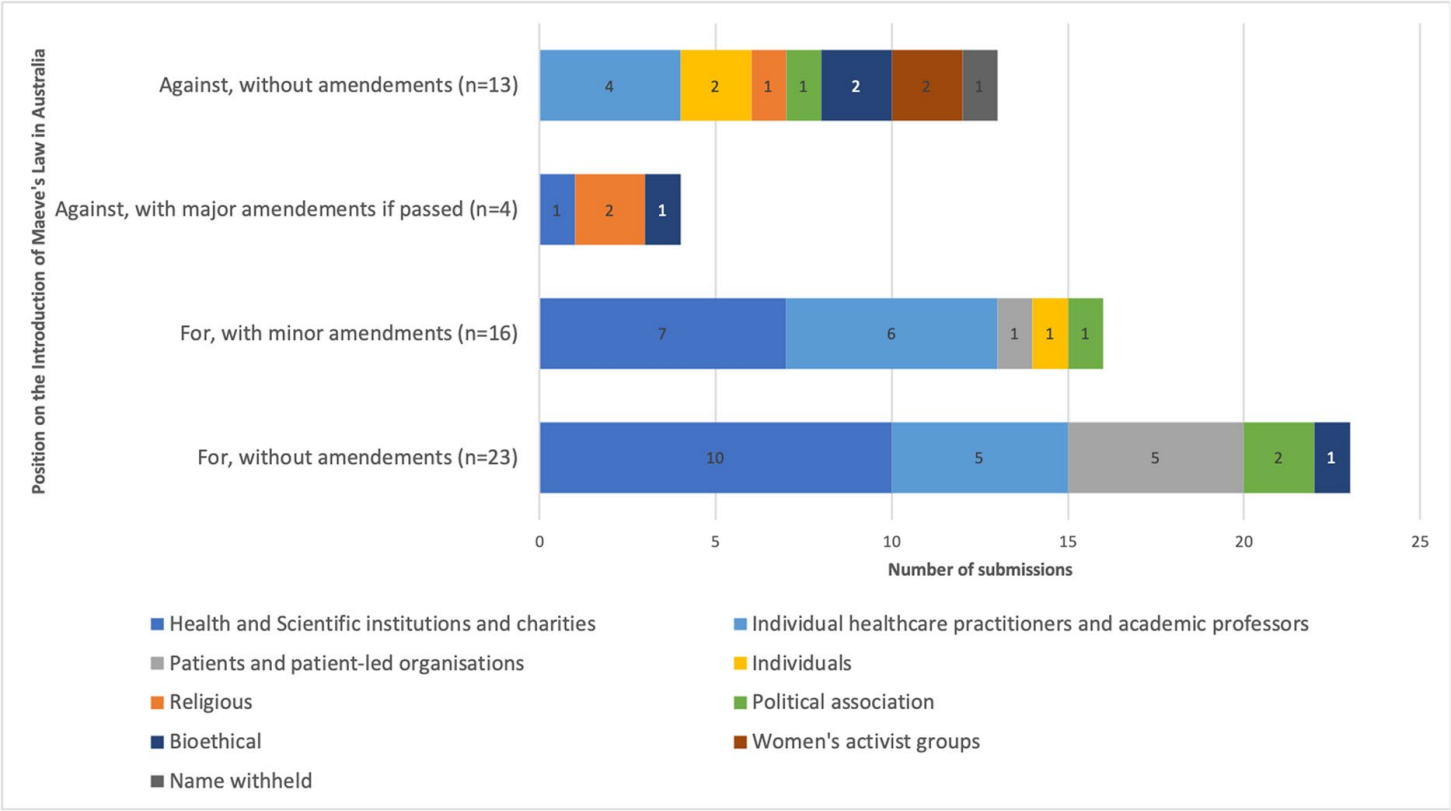
A comparative analysis of submissions for and against Mitochondrial Donation Law Reform (Maeve’s Law) Bill 2021

The majority of individuals, charities, or institutions lodging submissions in favour of the Bill had backgrounds in health and academia (Figure 1). Submissions from patients with mitochondrial disease and patient-led organizations were also largely in favour of the Bill. Those lodging submissions which oppose the Bill included religious organizations, women’s health groups, bioethical think tanks, political associations, individuals, and one anonymous submission.

Our analysis identified two overarching content categories: 1) arguments about whether Maeve’s Law should be passed by Australian parliament and 2) arguments about how Maeve’s Law should be implemented, each with subcategories based on category-specific arguments. Quotes from the submissions are included to illustrate the arguments raised.

## Views on Whether Maeve’s Law Should be Passed by the Australian Parliament

In the submissions discussing whether Maeve’s law should be passed, we identified six subcategories based on the focus of each submission: 1) the moral status of the human embryo; 2) the characteristics of the future child; 3) the implications for parents and/or families; 4) the safety and effectiveness of the technology; 5) the implications for society and future generations; and 6) legal precedence and international standards for mitochondrial donation.

### Moral Status of the Human Embryo

Several submissions raised concerns about mitochondrial donation based on the moral status of human embryos including violation of their human dignity. The lack of consideration of the rights and welfare of the unborn child was also presented as an argument against the Bill.

Experimentation on human embryos is problematic. Many legislatures recognise the moral status of the embryo and have banned the creation of human embryos for the purposes of experimentation. To do so represents instrumentalisation of the embryo for experimentation and destruction rather than implantation where it can fulfil its unique and dynamic destiny. (Submission 24)

### Characteristics of the Future Child

Many submissions identified the prevention of mitochondrial disease transmission to the child as a reason to pass Maeve’s law.

Importantly, by doing this transplant at the very early stage, the disease is cured. The children of the offspring of this procedure will themselves be free of mitochondrial disease. It would be eradicated forever in the individual and the family with no further need for treatment or management. (Submission 55)

Other submissions raised concerns regarding the potential unknown long-term health risks to the child and the psychological impact mitochondrial donation may have on the child’s sense of identity, either by virtue of being donor-conceived or through having three “biological” parents.

Having three genetic parents creates a real risk children will grow up struggling to find and understand their identity and heritage. (Submission 11)

The extent to which mitochondrial donation may influence the future characteristics of the child were also discussed. Some submissions argued that mitochondrial donation will make significant difference, while others negated this claim.

Just as nothing of importance changes about a person when she receives a heart transplant, nothing of moral importance changes when a cell receives a mitochondrial transplant. These are processes which differ merely in scale. (Submission 55)

Other submissions discussed mitochondrial donation in the context of the rights of the child born. Some claimed mitochondrial donation would violate a child’s right to a “natural” conception or to a “natural” human genome.

The proposed measures threaten the integrity of human genetic imprint which is owned by all, for all, and its protection is a universal human right. (Submission 47)

### Implications for Parents and Families Affected by Mitochondrial Disease

Submissions recognized the effects of mitochondrial donation on parents and family members as relevant to the ethics of mitochondrial donation, particularly to alleviate the suffering of affected families by enabling at risk individuals to have a genetically-related child free from mitochondrial disease.

Some submissions identified parental preferences as a motivating factor for the use of mitochondrial donation, and cast doubt as to whether parents have a right to a healthy genetically related child. Some who took this position held that prospective parents do not have a moral right to assistance in having a biological child, thus arguing that mitochondrial donation is merely a means of satisfying (less weighty) parental desires.

While sympathizing with families where mutated mtDNA exists, we would also like to note that there is no right to a biological child. It is difficult to see on what basis such a right would exist. (Submission 7)

Some submissions flagged that alternative options already exist for parents with a genetic predisposition to mitochondrial disease transmission and, as such, mitochondrial donation poses an unnecessary risk for mothers and children.

Other proven and less invasive options already exist, and are readily available to parents who want to have an mtDNA disease-free child e.g. adoption, fostering, egg donation, embryo donation, or pre-natal and pre‐implantation diagnosis and selective embryo transfer. Compared to these other methods, mtDNA transfer would increase the risk of a child suffering mtDNA disease. (Submission 36)

Yet, other submissions indicated that for some individuals with mitochondrial disease the options currently available may be unreliable or unsuitable.

For a subset of such women the currently available reproductive options are not suitable. (Submission 20)

Submissions also emphasized the importance of informed consent for all stakeholders given the clinical intricacies of mitochondrial donation.

Given the clinical and psychosocial complexities associated with mitochondrial donation, ANZICA strongly recommends that rigorous counselling and regulatory conditions (currently in place for third party reproduction) be applied. (Submission 46)

Of particular importance with regards to informed consent is that of parental autonomy. Several submissions praised Maeve’s Law, stating that it ensures that prospective parents receive genetic counselling about potential reproductive alternatives and the risks and uncertainties of this novel technology. Yet some submissions raised concerns that genetic counselling which offers mitochondrial donation as a preventative alternative may inadvertently coerce individuals to take up these novel techniques.

We know that women will feel pressured to adopt this technology. They will be told that the birth of children with mitochondrial disease is now preventable …

This will especially be the case for women who themselves have mitochondrial disease. The IVF industry will find a whole new group of customers, expanding its profit margins. (submission 37)

### Safety and Effectiveness of Mitochondrial Donation

A considerable number of submissions discussed issues relating to the safety of mitochondrial donation given the limited knowledge and information about success rates of live births at the time of legislating on which to assess clinical safety and efficacy. Some proposed that further preclinical research was needed to establish long-term health and safety outcomes.

However, at present, the relative safety and health impacts of these different methods are not known. This is a critical question to answer since it is well understood that physical manipulation can damage nuclear DNA and perturb epigenetics marks with severe consequences for the health of the offspring.

To address this, it is essential for more in-depth preclinical research to be conducted in one or more large animal models relevant to the human, to determine the relative safety and efficacy of these different technologies. (Submission 32)

Submissions also noted that these techniques can only be used to prevent disease transmission where the genetic mutation has occurred in the mitochondrial DNA and not where the genetic mutation is instead present in the nuclear DNA. Moreover, mitochondrial donation cannot be used to cure or treat individuals with existing disease, which some submissions held up as a serious limitation of the procedure.

Submissions also questioned the effectiveness of these techniques for preventing future transmission of defective genes in subsequent generations.

Nor is it known whether, given that up to 3% of the intending mother’s mitochondria is still likely to be passed on, the disease will rebound in future generations. (Submission 31)

One submission claimed that mitochondrial disease is strongly linked to environmental factors and thus, mitochondrial donation will be ineffective in preventing disease transmission without addressing these factors.

### Implications for Society and Future Generations

While some submissions discussed the potential for mitochondrial donation to prevent disease transmission to children born via mitochondrial donation and their descendant if successful; others raised concerns that heritable changes to the human genome that are incurred may have a negative influence on future generations.

Mitochondrial Donation is a form of genome modification … Importantly, this genome modification is a heritable germline manipulation, meaning that the children of any females conceived by Mitochondrial Donation will inherit the two female genomes, as mtDNA is passed through the female germline relatively unchanged. There is currently widespread support for the moratorium on genome modification in humans, yet Mitochondrial Donation would circumvent such bans. (Submission 32)

Others argued that mitochondrial donation should not be considered heritable gene-editing as it does not affect the nuclear DNA.

The proposed amendments would allow the use of mitochondrial donation for health and research purposes under a licence system administered by the NHMRC and using the process which has successfully regulated human embryo research in Australia for almost 20 years. What the amendments do not allow is alteration of genes either in the donor egg’s mitochondria or the parents’ nuclear DNA. This means that techniques for gene editing (like CRISPR) cannot be used to genetically modify the DNA of an embryo. (Submission 18)

In addition to these issues related to gene editing, many submissions were concerned with the use of mitochondrial donation for human enhancement and eugenics instead of for therapeutic purposes.

From a social perspective, some submissions noted that the introduction of mitochondrial donation may obscure the lines of social identity and human demographic history through mitochondrial DNA ancestral tracing.

MRT [Mitochondrial Replacement Therapy] changes kinship and ancestry in unpredictable ways. “ … genetic ancestry has become linked to important social and political debates over citizenship, social group boundaries, race, immigration policies, and exclusion.” By changing “matrilineal inheritance,” MRT interferes with the well-established understanding of genetic ancestry. (Submission 53)

One submission also raised concerns that mitochondrial donation may perpetuate social prejudice towards those who currently experience a genetic disability.

… Two women academics with disabilities discussing CRISPR gene editing to avoid disease and disability… “We have grave worries that the use of ‘genetic scissors’ will, in the future, cut people like us out of existence without others even noticing.” (Submission 37)

Finally, while some disputed whether the Australian public had been fully informed on the intricacies of the Bill, many submissions commended the extensive programme of community consultation which informed the drafting of Maeve’s Law. Thus, these submissions suggest that this Bill appropriately reflects broader societal opinion on the implementation of this novel technology.

The proposed legislation addresses the significant community support for legalising mitochondrial donation identified in previous rounds of consultation. (Submission 1)

### Legal Precedence and International Standards for Mitochondrial Donation

Several submissions also discussed the legal precedence of Maeve’s Law, both in the context of the U.K.’s mitochondrial donation law reforms, and in relation to Australia’s own Human Cloning laws.

According to the HFEA, the U.K. still has “limited evidence on risks and success rates”. However, information regarding the administration of the scheme in the U.K. to date is still a potentially valuable source of evidence regarding the practical outcomes of such a scheme in practice. Broadly, it seems that some available information and statistics about the U.K. scheme may exemplify the sorts of concerns we have raised above in these submissions. (Submission 24)

The process by which the equivalent U.K. regulations were passed was deeply flawed and is to be understood in relation to a particular national narrative. This process does not provide a good precedent and Australia should approach this question independently as though the regulations had no precedent elsewhere. (Submission 28)

Concerns were also raised regarding the notion that the introduction of Maeve’s Law provides a legal precedence which could expand the use of mitochondrial donation technology to other practices, such as subfertility and other forms of human genetic intervention.

If the Australian Parliament were to legalise this practice, it would open the door to other germ-line manipulations which until now have been condemned by the world. (Submission 23)

At a broader level, some submissions asserted that this legislation would put Australia out-of-step with current international opinion regarding mitochondrial donation, while others stated that these reforms are consistent with international standard and best practice in the area of genetic research.

The Bill’s reforms are consistent with international standards and best practice. For example, the International Society for Stem Cell Research, the preeminent global science organisation in this field, recently recommended that research and clinical use involving mitochondrial donation is permissible but only when subject to strict regulatory oversight and limited to patients at high risk of transmitting serious mitochondrial DNA-based diseases to their offspring.1 The licensing measures outlined in the Bill will provide the required oversight. (Submission 33)

## Views Towards How Maeve’s Law Should be Implemented in Australia

Category 2 comprised 4 subcategories regarding the implementation strategy of Maeve’s Law in Australia, including: 1) safety and efficacy of mitochondrial donation techniques, 2) regulatory oversight and safeguards in legislation, 3) equity and equitable access and 4) financial considerations.

### Safety and Efficacy of Mitochondrial Donation

Many submissions commend the Bill’s two-staged approach, which allows early access to mitochondrial donation while still ensuring a regulatory process for evaluating clinical safety and efficacy prior to its broader application into clinical practice.

In particular, it supports the use of licensing conditions to enable research, training and a clinical trial in Stage 1 to provide data and expertise needed to decide whether to progress to the introduction of mitochondrial donation into clinical practice. (Submission 48)

Some submissions referred to Australia’s scientific and medical expertise in novel reproductive technology. They expressed the opinion that Maeve’s Law also enables safe access to mitochondrial donation in a regulated environment and thereby may reduce the risk of individuals seeking unfettered services overseas.

Furthermore, there are unscrupulous operators who will offer mitochondrial donation less diligently in less regulated areas, employing lower standards and/or using these procedures for purposes unrelated to mitochondrial disease. Given this context, it is vital that the U.K., Australia and other interested countries work together to set high scientific, medical and regulatory standards for mitochondrial donation, thereby preventing the technology from being brought into disrepute. (Submission 27)

Several submissions supported eligibility criteria which initially restricts access to mitochondrial donation only to those at high risk of having a child with severe mitochondrial disease. However, given the complex nature of mitochondrial disease with regards to severity and risk of recurrence, it may be difficult to determine which individuals are considered “high risk.”

The Bill does not sufficiently address the parameters which will be used to identify suitable candidates for mitochondrial transfer. There are many difficulties with determining likely severity of mitochondrial disease, its manifestations and the risk of recurrence in future children. It is recommended that such a definition be included in the legislation. (Submission 24)

Others argued that there is limited benefit for mitochondrial donation in the community because of its stringent eligibility criteria.

How many families will actually be able to benefit from the techniques to have a child. In the U.K. so far, more than three years after the legalisation of the techniques, there has not yet been any child born through the use of mitochondrial donation, potentially demonstrating the specificity of the techniques and highlighting the barriers to its use. (Submission 56)

Maeve’s law also includes strict criteria for embryos in mitochondrial donation, restricting its use only in the context of mitochondrial donation, which many submissions considered appropriate.

… strongly believe that the control mechanisms and governance that are incorporated in this draft legislation will ensure that research and clinical implementation will solely benefit the families at risk of having children with these devastating diseases and prevent any potential misuse of the technique. (Submission 35)

Moreover, submissions mentioned that the Bill also ensures that children born via mitochondrial donation will not be subject to unnecessary long-term invasive monitoring, which many submissions supported. In contrast, some submissions were concerned the Bill lacks the necessary safeguards to protect the health of mothers with severe clinical manifestation of mitochondrial disease. They suggested that clinical evaluation and post-natal assessments of the mother’s health may be useful strategies to optimize outcomes in such cases.

[Maeve’s Law] however lacks the clinical safeguards necessary to guarantee the “health” of the very mother carrying the child … Without the input of clinical mitochondrial disease expertise into the evaluation and assessment of the mother before undergoing the technique, and then clinical surveillance antenatally, with appropriate follow-up postnatally, optimal outcomes for all are threatened. (Submission 41)

For this reason, many submissions agreed that timely access to mitochondrial donation is important given that delays may impact significantly on patient management and the time-sensitive nature of the mothers’ fertility and health.

It should be noted that long delays may have significant impacts on patient management and outcomes (e.g. long delays may have impacts on the mothers’ fertility and psychological health). (Submission 29)

Additionally, some submissions criticized the Bill for its promotion of egg donation, stating that these donation procedures are invasive and may incur potential long-term health consequences for donor women. In particular, a number of submissions raised concerns regarding the potential exploitation of women’s bodies for use in oocyte donation, given that a considerable number of oocytes would be required for research, training, and clinical use.

The sourcing of eggs for the procedure raises issues around the financial coercion of women … this leads to pressure from lobby groups to introduce payment for gamete donation, which leads to vulnerable women being financially coerced into undergoing a potentially life-threatening harvesting procedure. (Submission 12)

Therefore, many submissions advocated for the continuation of the ban on commercialization of egg donation to prevent the undue coercion and objectification of egg donors.

The current ban on commercialization of gamete donation should be maintained in order to avoid coercion of vulnerable females. (Submission 7)

Finally, some submissions provided specific recommendations to help improve efficacy of mitochondrial donation during the clinical phase. These included offering Preimplantation Genetic Diagnosis (PGD) testing as an alternative alongside mitochondrial donation in women who are at high risk of passing on mitochondrial disease, as well as arguments which dispute the amendments to include investment into germinal vesicle (GV) transfer.

### Regulatory Oversight and Safeguards in Legislation

#### Regulatory requirements and expertise

Maeve’s Law outlines stringent licencing and regulatory requirements while also acknowledging the importance of regulatory independence to prevent bias and ensure proper implementation of protocol. Importantly, families will require eligibility approval for use of mitochondrial donation but not individual licencing.

The Mito Foundation welcomes the fact that families will not require licenses but acknowledges the need for individual approval to ensure that mitochondrial donation only occurs in appropriate circumstances. (Submission 20 and submission 45)

These regulatory requirements also include the provision of specific Australian clinical expertise on licencing committees and training pathways. Many submissions supported this prerequisite given the complex and diverse health problems of this area of medicine.

The relevant committees and expert groups proposed to oversee mitochondrial donation are appropriate and proportionate. It is important however to ensure that the Licensing Committee either includes or has access to the support and advice of a clinician expert in mitochondrial medicine, given the broad range of medical issues that can be involved in patients with mitochondrial disease. (Submission 38)

#### Access to health-related information

Through the inclusion of confidentiality legislation and a donor register, Maeve’s Law protects the privacy of individuals involved, whilst still ensuring access to important health-related information for both personal and research purposes.

Maeve’s Law prioritises the privacy of families and children while at the same time ensuring that health monitoring of children will occur as will reporting of any adverse events. (Submission 16)

However, some submissions raised concerns that under this legislation, parents may refuse to participate in follow-up or share relevant information for privacy reasons which may skew data collected during the clinical trial phase.

Other sources also discuss other potential problems which may hinder long-term follow up in the U.K., including things such as the potential withdrawal of parental consent and the fact that it is not established whether follow-up should be left to physicians, parents or offspring. (Submission 24)

Additionally, access to donor information via a donor register is commended by many submissions as integral to protect the rights of the child.

We applaud the inclusion of the establishment of a donor registry within the bill to protect the rights of the offspring to knowledge of his or her biological origins, which is known to be important to donor offspring generally. It also allows the use of mtDNA for forensic purposes, should the need arise, where human remains are identified by comparing tissue to that of the (mtDNA donor) mother. (Submission 7)

#### Legal requirements and consent process for prospective parents

With regards to donor parental status, mitochondrial donors will not be considered parents, aligning Maeve’s Law with other Australian donor laws.

Consent counselling independence for prospective parents is also mentioned as an important issue to minimize the risk of coercion. However, some submissions outlined the difficulties of obtaining truly informed consent for an experimental process with limited research about long-term risk.

The issue of informed consent is difficult in situations where the prospective parents are required to understand complex scientific procedures which come with considerable risk. Independent counselling is required to allow fully informed consent. (Submission 24)

#### Ongoing monitoring and refinement of Maeve’s Law

A number of submissions highlighted the importance of ensuring ongoing monitoring and continuity of care during the transition between stages of implementation. However, some submissions raised concern about the lack of civil liability for the reporting of adverse events and long-term follow-up to govern the transition from research to clinical trial.

The Committee’s decisions to license this transition would not be public or subject to any review or right of appeal. Under the new laws, committee members, senior officials and politicians would also not be accountable or civilly liable for any of their decisions or actions that cause harm. (Submission 36)

Several submissions also indicated the need for future-proofing of legislation to ensure that the Bill is updated according to changes in novel techniques and new findings in the area.

Seven years between reviews is a long time in science, and new techniques (which may be more effective and/or safer) may emerge yet may not be able to be used in Australia. Is there a mechanism by which such flexibility as to recognising further new techniques might be incorporated over the time-span of this legislation? (Submission 49)

#### Syntax and semantics of Maeve’s Law

Many submissions also outlined the importance of scrupulous syntax in Maeve’s Law to ensure legislative rigor, including avoidance of ambiguity around sex selection terminology and use of gender-neutral terms.

VARTA recommends the Bill include language that is gender-neutral and inclusive of all people who wish to have a child in various ways. (Submission 30)

Similarly, several submissions discussed issues regarding reference terminology for mitochondrial donation, namely nuclear transplant, and whether mitochondrial donation may be considered analogous to organ transplantation or not.

Mitochondrial transfer is essentially the transplantation of healthy mitochondria to people with diseased mitochondria, just as we might transplant one kidney from a healthy person to another with kidney failure. This transplantation is at the microscopic scale: organelle transplantation. (Submission 55)

However, others argued that this terminology is misleading.

The word “donation” may mislead one into thinking that the procedures are simply extensions of existing practices such as organ transplantation and assisted reproductive technologies. This is not so.

Organ donation and transplantation has as its purpose the restoration of health in the person who is the recipient of the donated organ. “Mitochondrial donation” will not help anyone with mitochondrial disease: it will not cure or alleviate mitochondrial disease. In addition, and unlike organ transplantation, “mitochondrial donation” involves changes to the human genome that are potentially heritable. (Submission 31)

### Equality and Equitable Access to Mitochondrial Donation

Some submissions commented on the importance of equal access across States and Territories in Australia, as well as within priority populations, such as culturally and linguistically diverse (CALD) groups and those in rural and remote areas.

Ensure care and support is responsive to the specific needs of rural and remote communities and health services, Aboriginal and Torres Strait Islander people, those with CALD backgrounds, and other priority populations. (Submission 4)

Other submissions considered issues relating to the limited supply of donor oocytes given that egg donations are voluntary in Australia. This may restrict access to mitochondrial donation for research, training and clinical use.

Few submissions acknowledge the procurement of women’s eggs necessary for research and development of these technologies … Without women’s eggs there will be no mitochondrial donation program. (Submission 37)

Some suggested strategies to increase oocyte supply, including cryopreservation of oocytes and an oocyte donation programme.

Furthermore, equity was also discussed through the role of sex selection in mitochondrial donation in Australia. Some submissions argued that these techniques should be limited to use only in male embryos to prevent disease transmission, given the maternal inheritance pattern of mitochondrial DNA.

Maeve’s Law also allows for parents, following pre-treatment counselling and if safe and practicable, to choose to implant only male embryos. Given that mitochondrial DNA is passed only through the female line, this option would allay any potential risk that mitochondrial disease might reappear in future generations and, as such, is being offered to parents. (Submission 16)

Others disputed this, stating that there are ethical, social, and practical concerns which make sex selection of only male embryos problematic.

Sex selection is not permitted in the U.K. and would be difficult to justify with regard to the health of the female fetus or her risk of disease postnatally. Furthermore, determination of sex would currently require an additional manipulation of the embryo at an extremely early stage of development, potentially compromising viability of that embryo. (Submission 26)

### Financial Considerations

The Australian Federal Government has committed to funding the implementation of mitochondrial donation in research and clinical settings, which many submissions supported.

Prior to the introduction and debating of this bill in parliament, the Federal Government committed $4.4 million over four years in the 2021–22 Federal Budget (pp. [223–4]) to fund the implementation of mitochondrial donation in Australia’s research and clinical settings. (Submission 7)

However, some submissions appealed to concerns for distributive justice, arguing that investment in this novel technology could divert resources and funding away from other equally valuable ventures, including funding for a cure for mitochondrial disease or resource allocation for other heritable germline diseases.

… it’s very hard to accept the notion of using public funds to supposedly address this mitochondrial condition …The same resources could disproportionately achieve far more benefit put to those children presently suffering the disease … As well similarly the resources could be spent on the lives and processing of overseas refugee orphans for adoption. (Submission 39)

Several submissions also suggested specific recommendations including a cost-effective analysis of Maeve’s Law, introducing a centralized nation-wide approach to increase economies of scale and the effect of this legislation on commercialization of mitochondrial donation by the IVF industry.

The Bill could have the long-term effect of giving the private IVF industry unrestrained control over heritable human germline genetic transfers and manipulations, the future composition of the human gene pool and its evolution, and decisions on human genetic selection. (Submission 36)

## Discussion

To our knowledge, this is the only systematic analysis of the submissions received by the Senate committee. Although the majority of submissions supported the passage of Maeve’s Law, which was ultimately reflected in the passage of the Bill, they also reflect a diverse range of perspectives on the ethical issues raised by mitochondrial donation.

Our analysis of the Senate submissions supports previous findings that there is broad support for mitochondrial donation among the Australian community (Newson et al. 2019). Importantly, submissions from those with first-hand experience of mitochondrial disease, including clinicians and those with a family history of mitochondrial disease, were in strong support of this legislation. The most common arguments in support of Maeve’s law focused on the prevention of mitochondrial disease transmission to the unborn child, granting greater reproductive choice to affected individuals who may find current options unreliable or unsuitable, and protecting future generations from mitochondrial disease. The two-staged implementation strategy and rigorous licencing process were praised as effective measures to allow for early access to these techniques while research is ongoing.

Submissions which outlined arguments against the legislation either opposed the use of these techniques in general, and/or opposed aspects of the implementation strategy in Australia. Those opposed to the introduction of mitochondrial donation often appealed to rights-based moralities, arguing that such techniques could infringe on the presumed rights of the unborn child, as well as the rights of future generations. Opposition to Maeve’s was also grounded in the claim that there is no “right” to genetically related children, and thus mitochondrial donation only served to support morally insignificant parental preferences. Other submission opposed to mitochondrial donation cited the limited evidence regarding safety and efficacy of these novel techniques and focused on the risk of the technology.

Those opposed to the legislation also raised concerns regarding the cost-effectiveness of mitochondrial donation. Given the early stages of development into these novel technologies, it may be of greater societal impact to invest government funding and resources into other services such as adoption or funding for a cure for mitochondrial disease. They argued that resource allocation amongst other heritable germline diseases should also be considered during the implementation process of this legislation.

Despite ongoing research and debate into mitochondrial donation, there remains many unanswered questions. Since its approval in the United Kingdom in 2016, there is limited available evidence on the safety and effectiveness of mitochondrial donation in clinical practice. Freedom of information (FOI) requests published by the HFEA in January 2022 report that twenty-three mitochondrial donation procedures have been carried out since February 2017 (HFEA 2022). However, HFEA reports that between zero to five pregnancies have been achieved and between zero to five live births have been recorded following mitochondrial donation procedures (HFEA 2022). The lack of transparency regarding these findings suggests that perhaps these results have been less than satisfactory.

As a result, despite being the second country in the world to legalize mitochondrial donation, Australia has effectively drafted this legislation without including all the potentially available evidence on the safety and efficacy of mitochondrial donation in clinical practice. It is worth highlighting that Maeve’s Law outlines provisions for an independent review of operations two and three years after legislation has been passed, as well as a regular review process every seven years (The Parliament of the Commonwealth of Australia 2021). Such processes provide opportunities to incorporate clinical evidence as it comes to hand. It is important that at least some details of the monitoring both in the United Kingdom and in Australia are made publicly available to ensure that legislators can be apprised of whether these techniques are indeed effective and safe.

Interestingly, the themes we have identified in the submissions to the Australian Senate Community Affairs Legislation Committee overlaps largely—though not entirely—with the major themes in the ethics literature on mitochondrial donation (Newson, Wilkinson and Wrigley 2016). Major overlapping themes in the ethics literature include questions about how much importance we should place on opening new reproductive options for women at risk of transmitting mitochondrial diseases (Koplin et al. 2022), what kind(s) of consent should be required from mitochondrial donors (Schaefer 2018), and whether male embryos should be selected in order to minimize risks to future generations (Brandt 2018).

Interestingly, some major questions discussed in the ethics literature—such as whether mitochondrial donation should be used to facilitate “lesbian motherhood”(Cavaliere and Palacios-González 2018), whether mitochondrial donors should be permitted to be anonymous (Appleby 2018), and whether the practice of mitochondrial donation might unduly valorize the role of nuclear DNA compared to mitochondrial DNA (Sparrow, Mills and Carroll 2021)—were significantly less prominent in the submissions to the 2021 Australian Senate Committee than the wider bioethics literature. Other major themes of the Senate Committee submissions—for example, regarding the moral status of human embryos—have not been a major focus of the ethics literature.

This partial disconnect between the submissions we reviewed and the wider ethics literature suggests that the concerns that have received the most bioethical attention are not necessarily the ones that are the most politically salient. It also suggests that it is important for legislators and regulators to consider both public concerns and the broader bioethical discourse, since the issues raised in these two domains do not overlap completely (and both may identify factors that are morally and/or politically important.)

Legislators around the world should look to both the United Kingdom and Australia to inform their own legislative processes in this avant-garde area of medicine. It is important that despite the majority of views supporting legislation of mitochondrial donation, this may change if the techniques are found to be ineffective or unsafe.

In conclusion, the process of legalizing mitochondrial donation in Australia has ventilated a broad range of ethical issues by those invited to make a submission to the Senate Inquiry. While submissions generally expressed support for the legalization of mitochondrial donation in Australia, opposition to the Bill largely centred around technical recommendations regarding safety, accountability, and implementation strategy as well as calls for an international moratorium on heritable genome editing. Further consultation via a population-based questionnaire would help explore community opinions more broadly.

## Data Availability

Data from this study is available upon request.
